# Real-world Australian experience with tisagenlecleucel for relapsed/refractory diffuse large B-cell lymphoma—importance of pre-CAR-T optimization

**DOI:** 10.3389/fonc.2025.1633644

**Published:** 2025-08-19

**Authors:** Phoebe Joy Ho, Vinay Vanguru, Cale S. Burge, Rebecca Wayte, Christina Brown, Christian E. Bryant, Scott Dunkley, Derek McCulloch, Liane Khoo, James Favaloro, Anthony Jeffrey, Annie Solterbeck, Stephen Larsen, Edward Abadir

**Affiliations:** ^1^ Institute of Haematology, Royal Prince Alfred Hospital, Camperdown, NSW, Australia; ^2^ Faculty of Medicine and Health, University of Sydney, Sydney, NSW, Australia; ^3^ Statistical Revelations, Ocean Grove, VIC, Australia

**Keywords:** r/r DLBCL, CAR-T, real-world, tisagenlecleucel, relapsed, refractory, diffuse large B cell lymphoma

## Abstract

**Introduction:**

Up to 50% of patients with diffuse large B-cell lymphoma (DLBCL) relapse or are refractory to first-line therapy. Tisagenlecleucel, a CD19-directed chimeric antigen receptor (CAR)-T cell therapy, is approved for patients with relapsed/refractory (r/r) DLBCL in the third-line setting. Patients with r/r DLBCL treated with tisagenlecleucel in the real world have shown similar outcomes to those in clinical trials.

**Methods:**

We report a single-center real-world analysis of patients with r/r DLBCL treated with tisagenlecleucel.

**Results:**

As of December 31, 2024, 63 patients with r/r DLBCL had received tisagenlecleucel (median follow-up, 15 months). Cytokine release syndrome occurred in 89%; 95% were grades 1/2. Immune effector cell-associated neurotoxicity syndrome was reported in 17% (10/11 cases mild; one case grade ≥3). The overall response rate was 79%, with 60% complete response (CR). The median duration of response was 26.4 months. The median progression-free survival (PFS) was 14.6 months, and the overall survival (OS) was 15.4 months. Patients whose response at day 30 was CR had a 42% reduction in risk of progression compared with those who achieved partial response (PR). High lactate dehydrogenase (LDH) at infusion was associated with a higher risk of disease progression (hazard ratio [HR] 2.1) and death (HR 2.65) than normal LDH, with the risk for progression increased 3.3-fold in a multivariate model. Almost one-third of our patients who achieved CR/PR had normalized their LDH at the time of infusion from a previously elevated level, of whom 87% (13/15) had received bridging therapy. Lack of response to bridging was associated with an almost twofold increased risk of progression compared with the responsive patients (3.1 vs. 19.5 months; HR 1.9; 95% CI: 0.9–3.9, *P* = 0.08).

**Conclusion:**

We demonstrated in this analysis that the safety and efficacy of tisagenlecleucel in patients with r/r DLBCL in an Australian real-world setting were better than in the pivotal JULIET clinical trial and other registry studies. We also confirmed the importance of achieving early CR and normalizing the LDH levels at CAR-T cell infusion to reduce the risk of disease progression. Our results suggest that bridging therapy played an important role in optimizing outcomes by managing pre-CAR-T disease control.

## 1 Introduction

Diffuse large B-cell lymphoma (DLBCL) is the most common non-Hodgkin lymphoma, accounting for 30% to 50% of cases (1). Most patients with DLBCL respond well to first-line treatment combinations with rituximab, but up to 50% of patients have disease that is refractory to or relapses after first-line therapy (1, 2). Around 40% of these patients with relapsed/refractory (r/r) disease will respond to salvage chemotherapy and autologous stem cell transplantation, but 50% ultimately relapse despite transplant (2).

Tisagenlecleucel is a CD19-directed chimeric antigen receptor (CAR)-T cell therapy approved for use in adult patients with r/r DLBCL following two or more lines of prior therapy based on results from the pivotal JULIET trial (3, 4). The long-term results from JULIET (median follow-up, 40.3 months) showed that patients who received tisagenlecleucel had high rates of durable responses: the overall response rate (ORR) was 53%, the median progression-free survival (PFS) was 2.9 months, and the median overall survival (OS) was 11.1 months (5). The safety profile for tisagenlecleucel was manageable, with 22% of patients having experienced grades 3/4 cytokine release syndrome (CRS), and no deaths were attributed to tisagenlecleucel or CRS (4). No new safety concerns have been identified with longer follow-up (5).

Real-world data from patients with r/r DLBCL who received tisagenlecleucel in North America as collected by the Center for International Blood and Marrow Transplant Research (CIBMTR) suggest that the efficacy and safety outcomes are similar to or better than those reported in the JULIET clinical trial (4, 6). ORR was 59.5%, with a complete response (CR) rate of 44.5% with a median follow-up of 20.9 months. The median PFS was 4.1 months (95% CI: 3.5–4.9), the median OS was 16.4 months (95% CI: 14.6–21.0). Grade ≥3 CRS and immune effector cell-associated neurotoxicity syndrome (ICANS) were reported in 6% and 7.4% of patients, respectively (6). The real-world data are promising given that the patient population was more diverse than the population enrolled in the JULIET clinical trial and suggest that key learnings from the clinical trial have translated into better outcomes in the real-world setting. Here we report real-world outcomes for patients with DLBCL treated with standard-of-care tisagenlecleucel over a 4-year period at a single center in Australia. Within the treatment landscape of other CAR-T cell therapies for DLBCL, including axicabtagene ciloleucel and lisocabtagene maraleucel, real-world assessments of tisagenlecleucel as reported here are particularly relevant in many jurisdictions in which the use of tisagenlecleucel is crucial due to patient access.

## 2 Materials and methods

### 2.1 Study design

This single-center, real-world study included 63 consecutive patients with DLBCL who received tisagenlecleucel at Royal Prince Alfred Hospital in Sydney, Australia, between September 2020 and December 2024. The patients were followed until December 31, 2024, when outcomes data were censored. The main safety outcomes analyzed included the incidence and severity of CRS and ICANS. CRS and ICANS were graded using the American Society for Transplantation and Cellular Therapy (ASTCT) grading criteria (7) and represent the most severe grade reported. The serum lactate dehydrogenase (LDH) levels were measured at the time of enrollment and at the time of infusion. A normal LDH level in this study was defined as ≤250 U/L.

The efficacy outcomes were evaluated using CR, partial response (PR), progressive disease (PD), PFS, and OS. Positron emission tomography scan and Deauville criteria (8) at 1, 3, 6, and 12 months post-infusion were used to evaluate the response. ORR was defined as the best disease response (CR/PR) at any time after tisagenlecleucel infusion until disease progression or the start of new anticancer therapy. Duration of response (DOR) was defined as the time from tisagenlecleucel infusion to PD or death (from any cause) among patients who achieved at least PR. PFS was defined as the time from tisagenlecleucel infusion until disease progression or death from any cause. OS was defined as the time from tisagenlecleucel infusion until death from any cause. For patients who received bridging therapy, efficacy was also assessed according to response to bridging. Patients who relapsed within 12 months of the first chemoimmunotherapy regimen were compared with the rest of the cohort.

### 2.2 Statistical analysis

Descriptive statistics were used for baseline demographics and disease characteristics as well as incidence of adverse events. All time-to-event end points (DOR, PFS, and OS) were presented graphically overall and by subgroups representing key prognostic factors for patient outcomes (LDH at infusion [elevated/normal], early progression [yes/no], response to bridging [CR/PR vs. stable disease (SD)/PD], bridging therapy received [chemotherapy only, radiotherapy only]) using Kaplan–Meier survival plots. The median time (with 95% confidence limits, if possible) was obtained using the product limit estimator. To explore the effect of prognostic factors on outcomes, separate univariate Cox proportional hazards (PH) models were fitted and the HR obtained (with 95% confidence limits) and a *P* value to test the null hypothesis HR = 1 vs. HR ≠ 1. An exploratory multivariate Cox PH model was developed to explore the effect of each prognostic factor, adjusted for other factors in the model. Initially, multivariate models with various combinations of the four prognostic factors were considered, and the model with the best fit according to Akaike information criterion (AIC) was selected. Once the model was selected, the impact of including interaction terms was explored. From the final model, the HR (with 95% confidence limits) for each prognostic factor included was obtained, representing an adjusted HR.

## 3 Results

### 3.1 Baseline characteristics

Between September 2020 and December 2024, 63 patients with r/r DLBCL or DLBCL transformed from other subtypes received a single tisagenlecleucel infusion. The patient and disease characteristics are summarized in **Table 1**. The median age at the time of infusion was 71 years (range: 38–85). Most patients had r/r DLBCL (82.5%, *n* = 52); the remaining cases had DLBCL transformed from follicular or other indolent lymphoma (17.5%, *n* = 11). All patients had received two or more prior lines of therapy; 61.9% (*n* = 39) had received two prior lines, 27.0% (*n* = 17) had received three prior lines, 9.5% (*n* = 6) had received four prior lines, and 1.6% (*n* = 1) had received five prior lines; the median number of prior lines of therapy was two. At enrollment, 47.6% (*n* = 30) of patients had normal LDH levels (≤250 U/L), increasing to 61.9% (*n* = 39) at the time of CAR-T infusion. A total of 57 patients (90.5%) received bridging therapy before tisagenlecleucel infusion; 40% (*n* = 25) received radiotherapy alone, 43% (*n* = 27) received chemotherapy alone, 6% (*n* = 4) received both, and 2% (*n* = 1) received debulking surgery alone; 33% (*n* = 19) received rituximab-based therapy. Seven (11.1%) patients had pre-infusion grades 3–4 thrombocytopenia. The median follow-up of all patients was 15 months (95% CI: 10.9–19.2; range: 1–48).

**Table 1:** Baseline patient and clinical characteristics.

Category	Patients treated with tisagenlecleucel (*N* = 63)
Age at infusion, years, median (range)	71 (38–85)
Sex, *n* (%)	
Male	39 (61.9)
Female	24 (38.1)
Disease type, *n* (%)	
DLBCL	52 (82.5)
Follicular transformed to DLBCL	8 (12.7)
DLBCL transformed from other subtypes such as CLL and MZL	3 (4.8)
Number of prior therapies, *n* (%)	
2	39 (61.9)
3	17 (27.0)
4	6 (9.5)
5	1 (1.6)
Prior autologous HCT, *n* (%)	24 (38.1)
CNS involved at the time of infusion, *n* (%)	2 (3.0)
LDH at the time of enrollment, *n* (%)	
LDH normal^a^	30 (47.6)
LDH 251 to 375 U/L	24 (38.1)
LDH >375 U/L	9 (14.3)
LDH at the time of infusion, *n* (%)	
LDH normal^a^	39 (61.9)
LDH 251 to 375 U/L	18 (28.6)
LDH >375 U/L	6 (9.5)
Bridging therapy, *n* (%)	
Yes	57 (90.5)
Chemotherapy	27 (42.8)
Radiotherapy	25 (39.6)
Both	4 (6.3)
Surgery	1 (1.6)
No	6 (18.0)

a <250 U/L.

CLL, chronic lymphocytic leukemia; CNS, central nervous system; DLBCL, diffuse large B-cell lymphoma; HCT, hematopoietic cell transplant; LDH, lactate dehydrogenase; MZL, marginal zone lymphoma.

### 3.2 Safety outcomes

**Figure 1:**
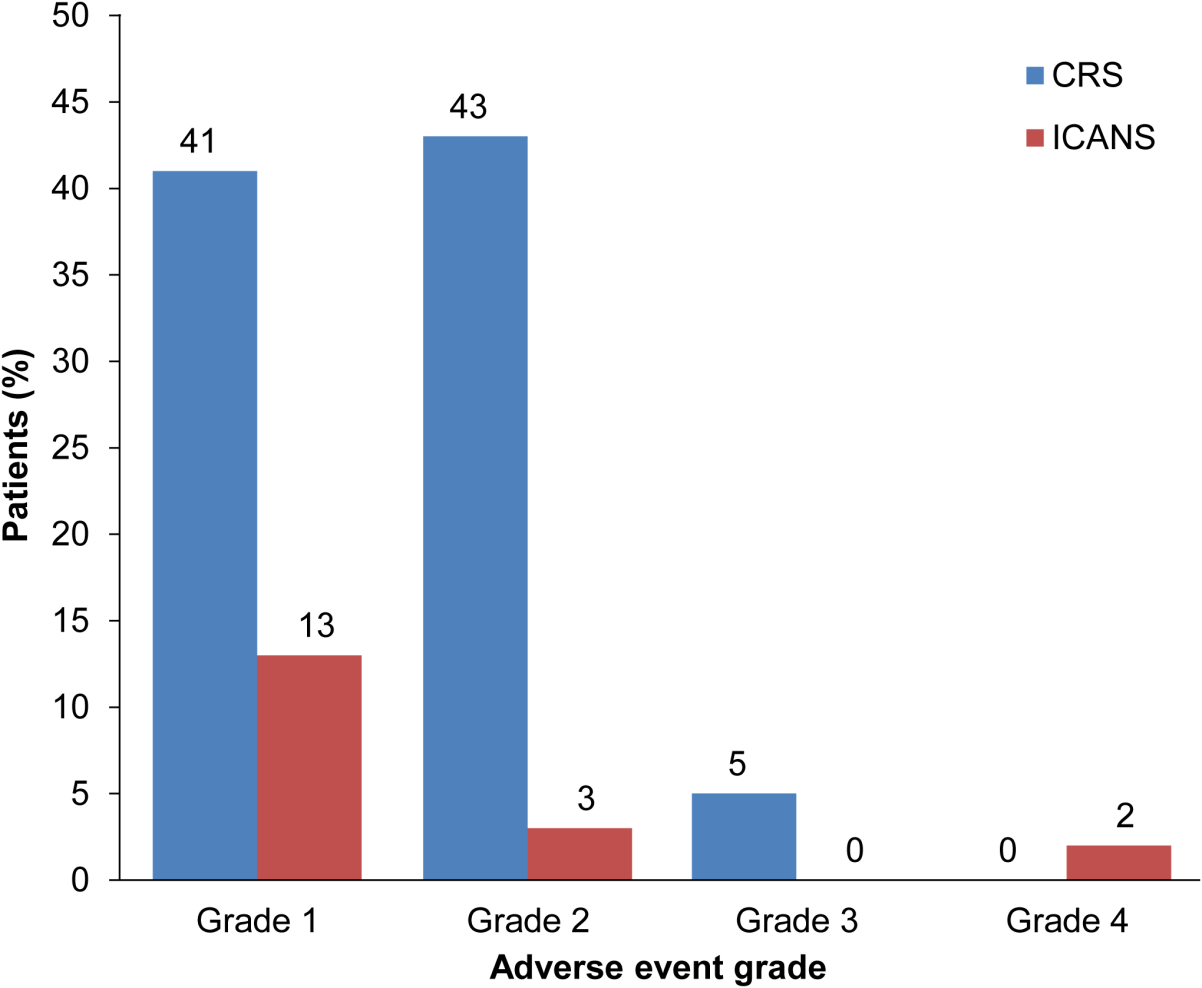
CRS and ICANS in patients treated with tisagenlecleucel. Rates according to severity of CRS (blue bars) and ICANS (red bars) in patients with r/r DLBCL treated with tisagenlecleucel. CRS, cytokine release syndrome; ICANS, immune effector cell-associated neurotoxicity syndrome.

Overall, CRS occurred in 88.9% (*n* = 56) of patients, comprising 84.1% (*n* = 53) with grades 1/2 CRS and 4.7% (*n* = 3) with grade 3; no grades 4/5 events were reported (**Figure 1**). ICANS was reported in 17.5% (*n* = 11) of patients, with 12.7% (*n* = 8) having grade 1, 3.2% (*n* = 2) grade 2, and 1.6% (*n* = 1) grade 4 (**Figure 1**). Among all tisagenlecleucel-infused patients, the median length of stay in the hospital after infusion was 17 days (range: 9–52).

A total of 50 patients (79.4%) were diagnosed with febrile neutropenia and treated empirically with intravenous antibiotics; all patients were being concurrently treated for CRS, and 6/50 patients had positive cultures with *Pseudomonas aeruginosa* (grade 3), *Clostridium difficile* (grade 2), COVID-19, *Klebsiella pneumoniae* (grade 4), *Campylobacter jejuni* (grade 4), and *Haemophilus influenzae* (grade 3). All patients recovered with antibacterial or antiviral treatments and supportive measures.

Cytopenias were reported following tisagenlecleucel infusion. Grades 3/4 neutropenias were reported in 20.0% (12/60) of patients at month 1, 12.0% (6/50) at month 3, 9.3% (4/43) at month 6, and 6.5% (2/31) at month 12. Grades 3/4 thrombocytopenia was reported in 28.3% (17/60) of patients at month 1, 14.0% (7/50) at month 3, 7.0% (3/43) at month 6, and 3.2% (1/31) at month 12.

The patients were followed for up to 48 months. During this time, secondary malignancies were reported in six patients (9.5%): three patients had myelodysplasia, two patients developed acute myeloid leukemia (one of these occurred following a subsequent allogeneic stem cell transplant), and one patient had breast cancer.

### 3.3 Efficacy outcomes

**Figure 2:**
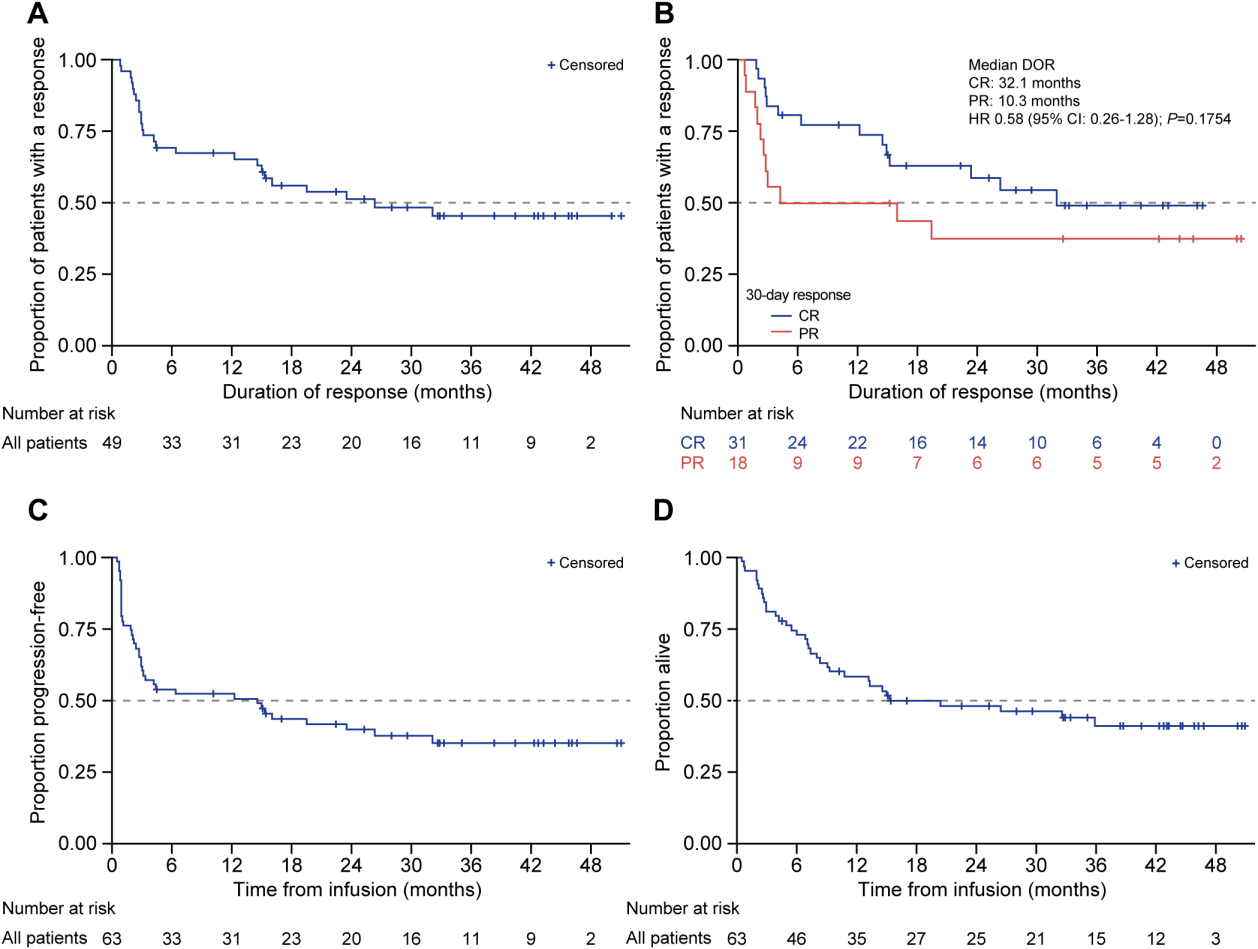
DOR, PFS, and OS in patients treated with tisagenlecleucel. Kaplan-Meier analysis was performed for **(A)** duration of response, **(B)** duration of response among responders (CR and PR), **(C)** PFS, and **(D)** OS in patients treated with tisagenlecleucel. The number of patients at risk at each time point is shown below the x-axis. CR, complete response; DOR, duration of response; HR, hazard ratio; OS, overall survival; PFS, progression-free survival; PR, partial response.

The median time from leukapheresis to infusion was 49 days (range: 36–348). A higher upper limit was due to a patient for whom leukapheresis was performed before entering a clinical trial of allogeneic CAR-T cells (prior to the eventual infusion of autologous tisagenlecleucel). Among all patients, ORR was 79.4% (CR: 60.3% [*n* = 38]; PR: 19.0% [*n* = 12]). In seven patients who had grades 3/4 thrombocytopenia prior to infusion, four of seven (57.1%) achieved CR and three of seven (42.9%) achieved PR. The median DOR was 26.4 months (**Figure 2A**) and was numerically higher among patients who achieved CR at day 30 (32.1 months [95% CI: 15.0–not reached (NR)]) vs. PR of 10.3 months (95% CI: 2.5–NR; HR 0.58 [95% CI 0.26–1.28]; *P* = 0.18; **Figure 2B**). The median PFS was 14.6 months (95% CI: 3.0–26.4; **Figure 2C**), and the median OS was 15.4 months (95% CI: 8.3–NR; **Figure 2D**). The PFS and OS at 12 months were 52.3% and 58.3%, respectively, with OS at the data cutoff date of 41.1% (median follow-up: 14.8 months).

**Figure 3:**
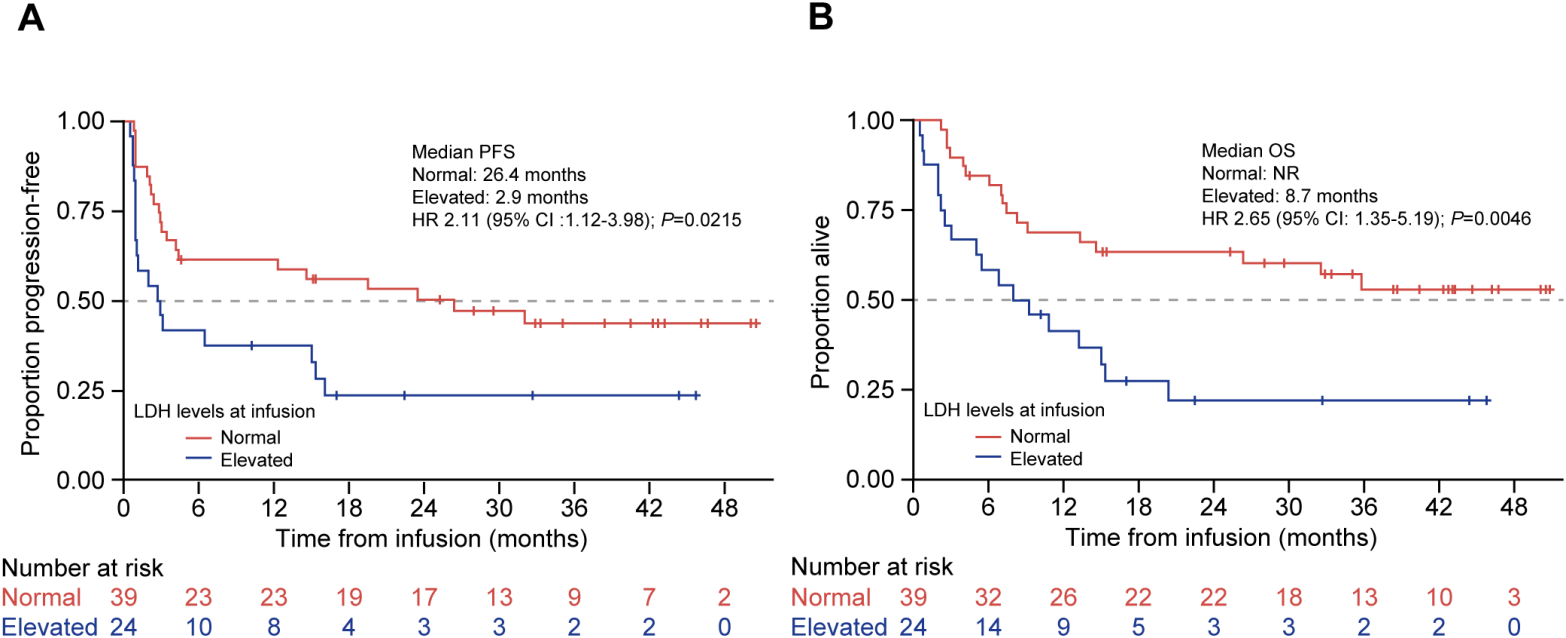
PFS and OS by preinfusion LDH levels in patients treated with tisagenlecleucel. Kaplan-Meier curves for **(A)** PFS and **(B)** OS of patients treated with tisagenlecleucel were analyzed and compared between patients with normal (red line) and elevated (blue line; >250 U/L) LDH levels. The number of patients at risk at each time point is shown below the x-axis. HR, hazard ratio; LDH, lactate dehydrogenase; NR, not reached; OS, overall survival; PFS, progression-free survival.

The median PFS among patients with normal LDH (≤250 U/L) at infusion was significantly longer (26.4 months [95% CI: 3.4–NR]) compared with patients with high LDH (>250 U/L) (2.9 months [95% CI: 0.9–15.4]; HR 2.11 [95% CI: 1.12–3.98]; *P* = 0.02; **Figure 3A**). The median OS for patients with normal LDH at infusion was not reached (95% CI: 13.3–NR) vs. 8.7 months (95% CI: 2.6–15.4) for patients with high LDH (HR 2.65 [95% CI: 1.35–5.19]; *P* = 0.0046; **Figure 3B**). Among patients whose disease responded to tisagenlecleucel therapy (CR/PR; *n* = 50), the majority (*n* = 34, 68.0%) had normal LDH levels at infusion—they were always normal in 19 (38.0%) patients, had normalized at the time of infusion in 15 (30.0%) patients, were persistently elevated before and at infusion in nine (18.0%) patients, and were increased from normal to elevated in seven (14.0%) patients. Of the 15 patients whose LDH had normalized from enrollment to the time of CAR-T infusion, 13 had received bridging therapy—radiotherapy in five, chemotherapy in six, and both modalities in two patients.

**Figure 4:**
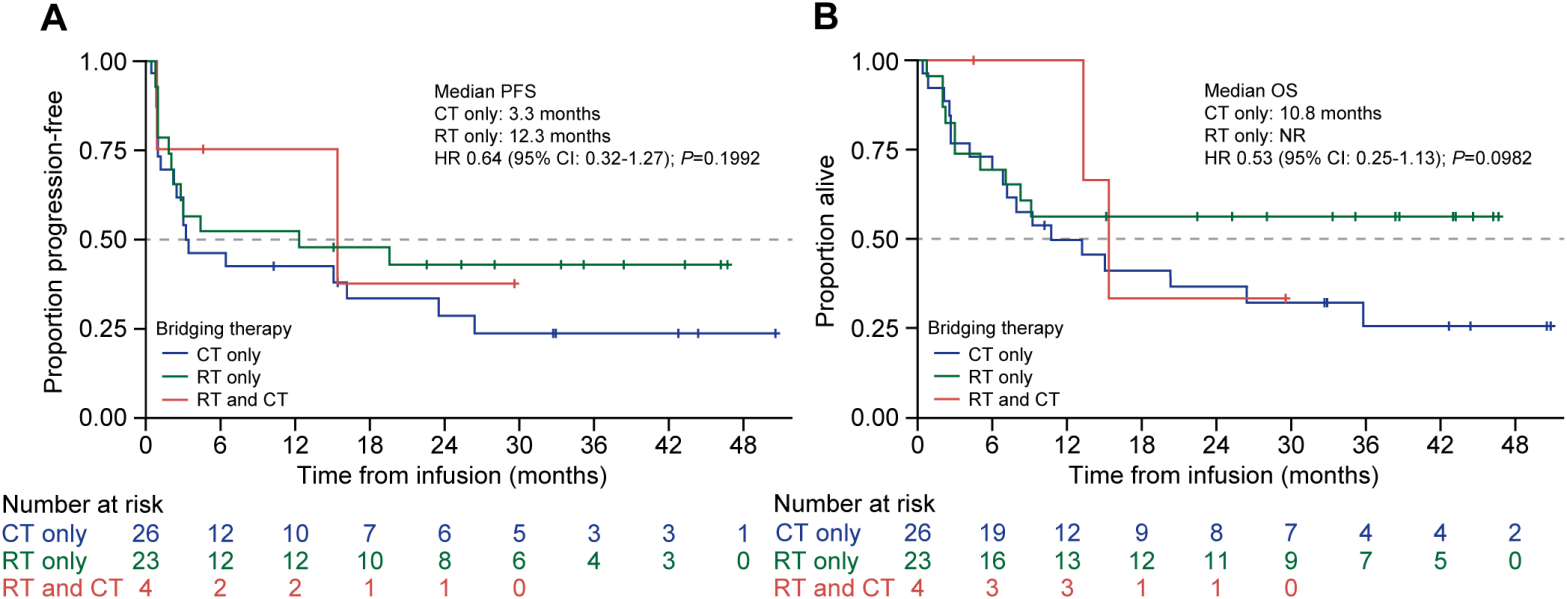
PFS and OS by type of bridging therapy used in patients treated with tisagenlecleucel. Kaplan-Meier curves for **(A)** PFS and **(B)** OS of patients treated with tisagenlecleucel were analyzed and compared between patients given chemotherapy alone (blue line), radiotherapy alone (green line), or both (red line) as bridging therapy prior to tisagenlecleucel infusion. The number of patients at risk at each time point is shown below the x-axis. CT, chemotherapy: HR, hazard ratio; NR, not reached, OS, overall survival; PFS, progression-free survival: RT, radiotherapy.

Among patients who received bridging therapy (57/63, 90.5%), the median PFS for patients who underwent chemotherapy alone and radiotherapy alone was 3.3 months (95% CI: 1.1–23.5) and 12.3 months (95% CI: 2.0–NR), respectively (*P* = 0.20), whereas five patients who received both modalities had PFS of 15.4 months (95% CI: 0.9–NR) (**Figure 4A**). The median OS for patients who received chemotherapy alone was 10.8 months (95% CI: 6.1–35.8), for patients who received radiotherapy alone it was NR (95% CI: 5.0–NR) (*P* = 0.1), and for the five patients who received both it was 15.4 months (95% CI: 13.3–NR) (**Figure 4B**).

**Figure 5:**
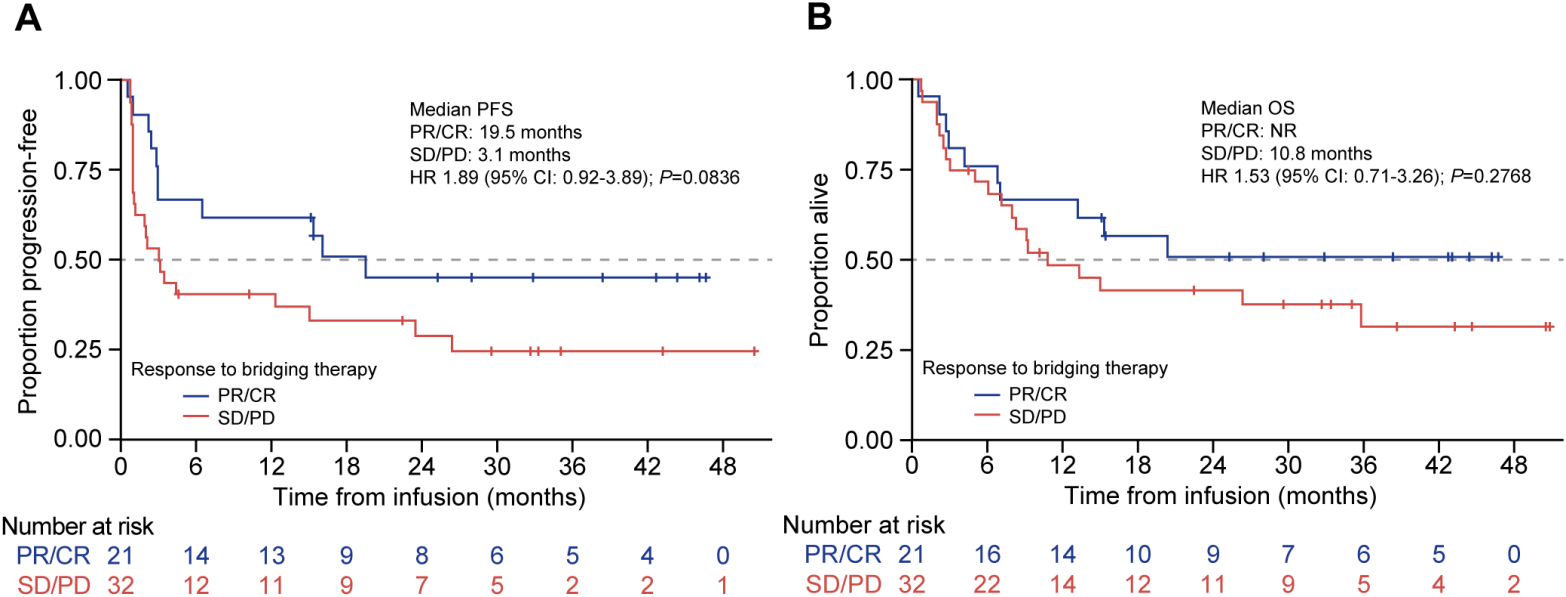
PFS and OS by response to bridging therapy in patients treated with tisagenlecleucel. Kaplan-Meier curves for **(A)** PFS and **(B)** OS of patients treated with tisagenlecleucel who were given bridging therapy and had a response (blue line) or no response (red line). The number of patients at risk at each time point is shown below the x-axis. CR, complete response; HR, hazard ratio; NR, not reached: PD, progressive disease; PFS, progression-free survival; PR, partial response; OS, overall survival; SD, stable disease.

Analysis by response to bridging showed that the patients who achieved CR or PR to bridging therapy showed improved median PFS compared with those who had SD or PD, but this did not reach statistical significance, which was likely due to the small numbers (19.5 months [95% CI: 3.0–NR] vs. 3.1 months [95% CI: 1.0–15.0]; HR 1.89 [95% CI: 0.92–3.89]; *P* = 0.08; **Figure 5A**). The median OS was also numerically superior for the patients who responded, which was NR (95% CI: 6.9 months–NR) vs. 10.8 months (95% CI: 6.1–35.8) for SD/PD; HR 1.53 (95% CI: 0.71–3.26); *P* = 0.28 (**Figure 5B**).

**Figure 6:**
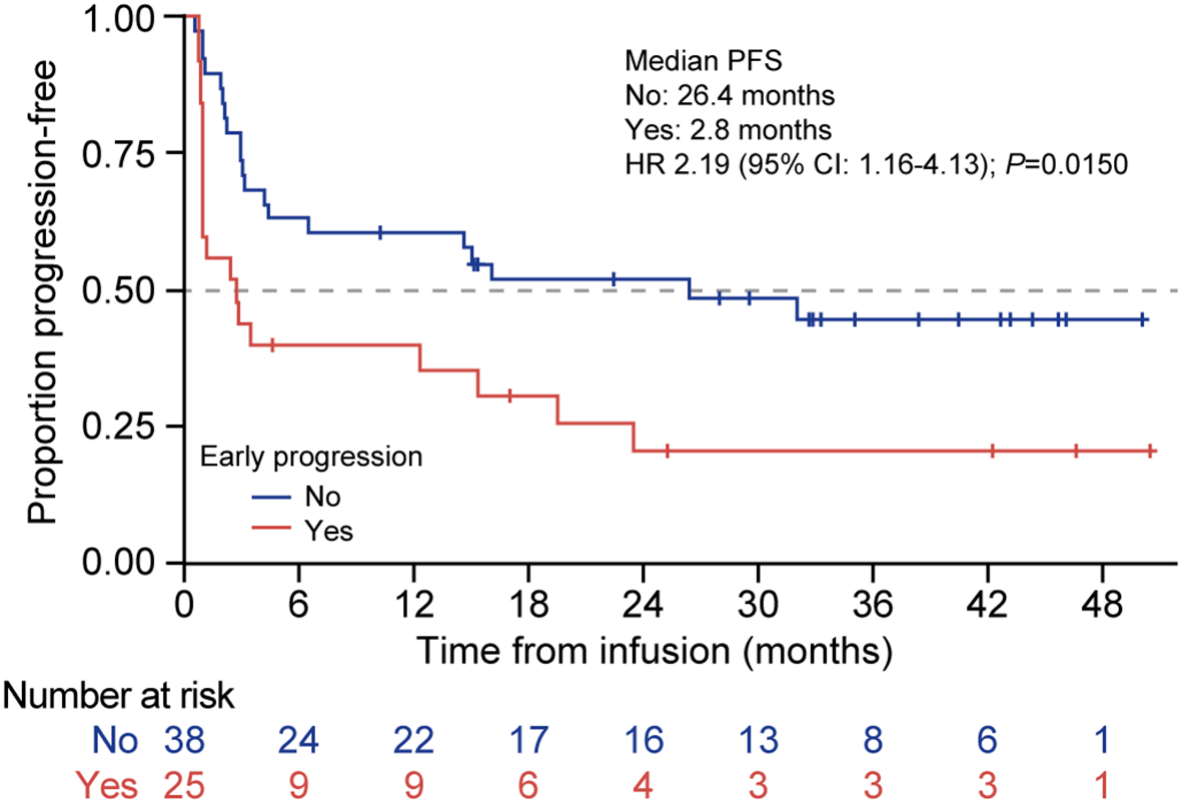
PFS according to early vs. late disease relapse from first-line therapy in patients treated with tisagenlecleucel. Kaplan-Meier curves for PFS of patients treated with tisagenlecleucel who had disease relapse <12 months after first-line immunochemotherapy (red line) compared with those who did not experience early disease relapse after initial therapy (blue line). The number of patients at risk at each time point is shown below the x-axis. HR, hazard ratio; PFS, progression-free survival.

The patients whose disease relapsed within 12 months of the initial chemoimmunotherapy had a significantly worse median PFS compared with those with later relapse (2.8 months [95% CI: 0.9–15.4] vs. 26.4 months [95% CI: 4.2–NR], respectively; HR 2.2 [95% CI: 1.2–4.1]; *P* = 0.02; **Figure 6**).

**Figure 7:**
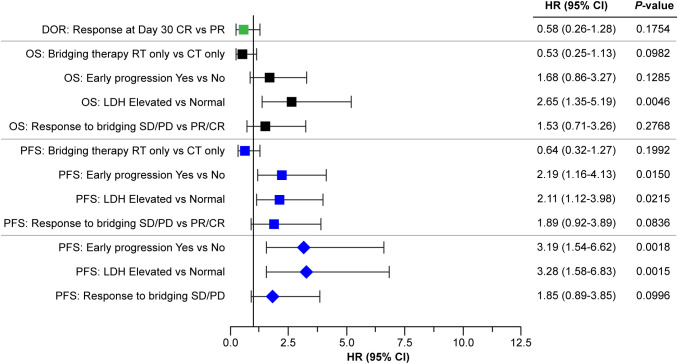
Forest plot of efficacy outcomes by response at day 30, LDH levels, primary refractoriness, and response to bridging. Squares indicate the HRs for the parameters analyzed by univariate analysis, diamonds indicate parameters analyzed by the multivariate model. CR, complete response; CT, chemotherapy; DOR, duration of response; HR, hazard ratio; LDH, lactate dehydrogenase; OS, overall survival, PD, progressive disease; PFS, progression-free survival; PR, partial response; RT, radiotherapy, SD, stable disease.

An exploratory multivariate model was developed to assess the impact of prognostic factors (LDH, early progression to initial therapy, response to bridging, and bridging modality) on PFS. A three-factor model, including LDH (elevated vs. normal), early progression (yes vs. no), and response to bridging (CR/PR vs. SD/PD), was selected based on the AIC (AIC is an indication of model fit). The inclusion of interaction terms between these three factors was explored, but none improved the model fit. The results from this three-factor model demonstrated that LDH level at infusion (elevated vs. normal, adjusted HR 3.3; 95% CI: 1.6–6.8; *P* = 0.0015) and early progression (yes vs. no; adjusted HR 3.2; 95% CI: 1.5–6.6; *P* = 0.0015) had a marked impact on PFS, having adjusted for the other two factors in the model. Response to bridging therapy (no response [SD/PD] vs. response [PR/CR]) demonstrated a 1.9-fold increase in the risk of progression in patients who did not have a response to bridging therapy compared with those who responded (adjusted HR 1.85; 95% CI: 0.89–3.85; *P* = 0.10). This is clinically relevant although not statistically significant, which is likely due to the small sample size (**Figure 7**).

## 4 Discussion

This report represents a single-center experience among patients receiving tisagenlecleucel for r/r DLBCL in Australia and adds to the body of real-world evidence in this patient population (6, 9–14). Although the retrospective nature of this observational study and the smaller sample size and shorter follow-up than the JULIET trial and CIBMTR real-world analysis are limitations, we showed that the efficacy and safety in our cohort were better than in the JULIET trial, registry, and other real-world studies. In addition, the description of the results of one CAR-T product from a single institution, being one of the first institutions to adopt CAR-T therapy in our jurisdiction, is also valuable to benchmark against efficacy in other jurisdictions such as the CIBMTR data and highlight possible geographical differences.

Our cohort of patients was older overall than in the JULIET trial and CIBMTR study but experienced similar or better efficacy and safety outcomes. The 79.4% ORR in our study was better than that in the JULIET trial (52%) and the CIBMTR study (59.5%) (4, 6). Other real-world studies showed lower 3-month ORR (~40%) than that reported for the CIBMTR registry (12, 13). Our CR rate of 60.3% was also substantially better than that of the CIBMTR study (44.5%) and other real-world studies (35%-39%) (6, 12, 13). Interestingly, the finding in the JULIET study that severe thrombocytopenia pre-infusion was a significant risk factor for reduced response was not seen in our cohort, noting, however, our small numbers: all seven patients pre-infusion grades 3/4 thrombocytopenia achieved CR or PR. The median PFS (14.6 months) and OS (15.4 months) reported here were longer than the median PFS (2.9 months [95% CI: 2.3–5.2]) and OS (11.1 months [95% CI: 6.6–23.9]) reported for patients in JULIET with 40.3 months of follow-up (5). These outcomes were also improved especially for PFS and similar for OS compared with real-world results reported by CIBMTR (median PFS: 4.2 months; median OS: 16.4 months) and better than another real-world study (median PFS: 3.0 months; median OS, 11.8 months) (6, 13). As in previous studies, we showed a good correlation between the level of response with PFS. Patients whose response at day 30 was CR had a 42% reduction in risk of progression compared with patients whose day 30 response was PR. Furthermore, we observed lower rates of high-grade CRS and ICANS than in the JULIET trial and the CIBMTR real-world analysis. Grades 3/4 CRS occurred in 4.7% of our patients compared with 22% in JULIET and 6% in CIBMTR (4, 6). One case (1.6%) of grade ≥3 ICANS event was reported in our cohort compared with 11% in JULIET and 7.4% in CIBMTR (5, 6). Although the overall rate of CRS was high (89%), it was predominantly mild (84%). It is also noted that 79.4% (50/63) of the patients in our cohort were treated for febrile neutropenia, and all were concurrently treated for CRS due to the possible clinical overlap. In our effort not to underestimate CRS, it is likely that a sizeable proportion of the patients considered to have CRS may have fever due to febrile neutropenia. Our rate of secondary malignancy of 9.5% (*n* = 6/63) was also comparable with previous studies, including 7% from the JULIET study (15), with a similar follow-up.

The improved efficacy and safety outcomes in patients with DLBCL treated with tisagenlecleucel in our study may be a result of the high proportion of patients (61.9%) who had normal LDH (≤250 U/L) at the time of infusion. Previous results indicate that normal LDH levels at baseline were associated with improved PFS and OS (9, 16)—for example, high baseline LDH levels, defined as more than twofold of the upper limit of normal (ULN), were associated with worse efficacy outcomes in the JULIET trial (5, 16). High baseline LDH was associated with nonresponse (odds ratio 5.59; 95% CI: 1.43–21.92) and lower ORR (19.0%) compared with patients with baseline normal LDH (≤ULN; 52.7%) (5, 16). An analysis of real-world data from the CIBMTR registry also reported that normal LDH levels prior to infusion were associated with improved PFS and OS (9, 16). Consistent with these results, our study showed significantly worse PFS and OS in patients with elevated LDH at infusion, with 2.1- and 2.7-fold increased risk of progression and death, respectively, and the risk of progression increased by 3.3-fold in a multivariate model adjusting for early progression to initial therapy and response to bridging.

Bridging therapy can be used to control disease progression in patients with r/r DLBCL while waiting for CAR-T cell manufacturing to debulk the disease and to reduce the risk of CAR-T cell therapy-associated toxicities (e.g., CRS and ICANS) (17). Bridging therapy was allowed in several clinical trials evaluating various CAR-T cell products, but the data did not show a clear relationship between bridging therapy and clinical outcome (4, 17–21). Most patients in the JULIET trial (92%) received bridging therapy, and an analysis of seven patients who achieved CR after bridging therapy showed that five of seven patients remained progression-free at >12 months post-infusion (4, 21). The need for bridging therapy has often been associated with worse long-term CAR-T cell therapy outcomes, as patients who require bridging therapy often have unfavorable disease characteristics, such as elevated LDH or bulky disease (22). Among patients who require bridging therapy, outcomes can vary by the type of therapy used (22). Radiotherapy has been shown to be an effective bridging option for patients with rapidly progressing disease (23) and has been shown to have a synergistic effect when combined with CAR-T cell therapy (24). In this study, the median PFS of patients who underwent radiotherapy alone for bridging was 15.4 months, and it was 3.3 months for chemotherapy (*P* = 0.20), which is likely attributed to our practice of utilizing radiotherapy in more localized relapses and chemotherapy for extensive disease.

Administering bridging therapy to debulk the tumor and normalize LDH prior to infusion was also seen in our study. Of 15 patients who normalized their LDH from enrollment to infusion and achieved a response, 13 had received bridging therapy. A lack of response to bridging therapy conferred almost twofold risk of progression by univariate analysis (PFS: 3.1 vs. 19.5 months; HR 1.89; *P* = 0.08). This is clinically relevant although not statistically significant, which is likely due to interactions with LDH levels and early progression as shown in the multivariate model and the small sample size (**Figure 7**). The high proportion of patients in our study who had normal LDH at the time of infusion, with almost one-third of responsive patients normalizing their LDH before CAR-T infusion with bridging therapy, supports the observation that reducing disease burden systemically prior to CAR-T cell infusion may be the cause of our higher response rates and durability (25). It is worth noting that the percentage of patients who had a normal LDH had increased from 47.8% at enrollment to 61.9% at the time of CAR-T infusion in our study. Further investigation into the role of bridging therapy to reduce disease burden and optimize baseline risk factors, such as high LDH levels prior to CAR-T cell therapy, is warranted.

### 4.1 Strengths and limitations

Our cohort of 63 consecutive patients treated by tisagenlecleucel represents a substantial cohort of r/r DLBL patients receiving a uniform treatment in the real world. Our results show satisfactory efficacy and safety that are superior to previous trials and real-world data and suggest the importance of optimizing disease status by using bridging therapy as a strategy to improve outcomes. As noted earlier, despite the approval of other CAR-T cell therapies for DLBCL, an understanding of the impact of tisagenlecleucel is particularly relevant in jurisdictions for which it is crucial for patient access to CAR-T cell therapy. Limitations include the retrospective, single-center analysis of the study. Due to the retrospective nature of this observational study, a more detailed analysis of adverse events would likely be incomplete depending on the accuracy of medical records, whereas CRS, ICANS, infection, cytopenia, and secondary malignancy were documented in structured proforma. For this reason, our report of safety was centered on these crucial features of safety outcomes rather than a more extensive listing.

In summary, our real-world study adds to a growing body of data that confirms and extends the efficacy and safety profiles of tisagenlecleucel reported in the pivotal clinical trial. It is likely that improvements in disease debulking prior to infusion and clinical experience in managing CRS and ICANS have contributed to the real-world gains reported to date. Future analyses may provide further insight into strategies to optimize the use of tisagenlecleucel in patients with r/r DLBCL.

## Data Availability

The raw data supporting the conclusions of this article will be made available by the authors, without undue reservation.
